# β-LAPachone is renoprotective in streptozotocin-induced diabetic mice via regulating the PI3K/Akt/mTOR signaling pathway

**DOI:** 10.22038/ijbms.2021.55565.12422

**Published:** 2021-05

**Authors:** Davoud Sanajou, Saman Bahrambeigi, Somayeh Aslani

**Affiliations:** 1 Department of Biochemistry, School of Medicine, Tabriz University of Medical Sciences, Tabriz, Iran; 2 Department of Biochemistry, School of Medicine, Hamedan University of Medical Sciences, Hamedan, Iran

**Keywords:** Akt, β-LAPachone, Diabetic nephropathy, mTOR, PI3K

## Abstract

**Objective(s):**

β-LAPachone (B-LAP) is a natural product with established anti-inflammatory properties. In this study, we investigated the protective potential of B-LAP against diabetic nephropathy in streptozotocin (STZ) induced diabetic mice.

**Materials and Methods:**

Diabetes induction in mice was carried out by a single intraperitoneal injection of STZ. 2.5 mg/kg/day and 5 mg/kg/day doses of B-LAP were administered orally for twelve weeks and renal histoarchitecture, caspase-3, tumor necrosis factor-alpha (TNF-α), malondialdehyde (MDA), glutathione peroxidase (GPX), as well as urinary nephrin and neutrophil gelatinase-associated lipocalin (NGAL) were evaluated. Additionally, kidney levels of PI3K, phosphorylated (p)-Akt, p-mTOR, p-CREB, and SIRT1 were assessed in the present investigation.

**Results:**

5 mg/kg B-LAP significantly decreased urinary excretions of nephrin and NGAL. It also mitigated renal TNF-α and MDA levels and simultaneously improved GPX activities. 5 mg/kg B-LAP improved renal function in diabetic mice as indicated by elevated values of creatinine clearance. While B-LAP elevated renal levels of SIRT1, it alleviated PI3K, p-Akt, p-mTOR, and p-CREB levels in the kidneys of diabetic mice.

**Conclusion:**

Collectively, these findings suggest B-LAP as a potential renoprotective agent in STZ-induced diabetic mice probably via modulating the PI3K/Akt/mTOR pathway.

## Introduction

Diabetic nephropathy (DN) is the main cause of end-stage renal disease (ESRD) across the world, which develops in one-third of both type 1 and type 2 diabetic patients (1). Strict glycemic control, as well as blockade of the renin-angiotensin axis, are the two main management modalities for DN and despite these measures, a considerable number of DN patients progress to ESRD, underlining the importance of developing novel adjunctive therapeutic agents for DN (2). 

DN has always been considered the result of relentless hyperglycemia and constant glomerular hypertension; however, the marked contribution of chronic inflammation to the pathophysiology of DN has now been well understood (3). Inflammation, as well as increased generation of reactive oxygen species (ROS), initiate various intracellular signaling pathways such as JAK/STAT, NF-κB, MAPK, and TGF-β1/Smad3 which collectively deteriorate renal function in diabetic kidney disease (4). While the conventional non-steroidal anti-inflammatory agents, i.e., cyclooxygenase inhibitors have failed to retard DN progression, the targeted inhibition of the inflammatory mediators including monocyte chemo-attractant protein 1 (MCP-1), C-C chemokine receptor type 2 (CCR-2), and Janus kinase 1/2 (JAK1/2) in phase 2 clinical trials have shown substantial renoprotective effects in DN patients (5).

β-LAPachone (B-LAP), the natural agent derived from the Lapacho tree, *Handroanthus impetiginosus*, with anti-inflammatory properties, is able to down-regulate interleukin 1β (IL-1β), IL-6, and tumor necrosis factor-alpha (TNF-α) levels via modulating NF-κB and MAPK/ERK signaling pathways *in vitro* (6). Meanwhile, B-LAP suppresses collagen production via modulating the TFGβ-Smad signaling pathway (7). Newer classes of protective agents capable of delaying DN progression such as FPS-ZM1, empagliflozin, and liraglutide all act principally by repressing the inflammation/oxidative stress/apoptosis triad in the diabetic kidneys via their pleiotropic effects (8-10). Moreover, B-LAP renders protection against doxorubicin-induced nephrotoxicity in mice via modulating the AMPK pathway (11).

The present investigation aimed to examine potential renoprotective effects of B-LAP in streptozotocin (STZ)-induced diabetic mice and to evaluate its impact on the PI3K/Akt/mTOR pathway in the kidneys.

## Materials and Methods

***Chemicals***

The following commercially available chemicals were obtained: B-LAP (Cayman Chemical, Ann Arbor, MI, USA) and STZ (Santa Cruz, Dallas, TX, USA).

***Experimental design***

Thirty male C57BL/6 mice were obtained from the Pasteur Institute of Iran. Diabetes induction was done by injecting a 50 mg/kg dose of STZ dissolved in citrate buffer (pH: 4.5) (12). Induction of diabetes was confirmed by assessing the tail-blood glucose levels 48 hr after STZ injection and mice with glucose levels over 250 mg/dL were included in the study (12). All animal experiments and procedures were conducted with adherence to Guidelines for the Care and Use of Animals in Scientific Research issued by the Research Council of Tabriz University of Medical Sciences . Animal Ethics Committee of Tabriz University of Medical Sciences approved the protocol of the study (IR.TBZMED.REC.1397.253).

Mice were assigned into 5 groups of 6 animals per each; 1- sham receiving B-LAP vehicle, i.e., 0.05% DMSO; 2- diabetic control mice (Diab); 3- Diab + low dose B-LAP (2.5 mg/kg/day); 4- Diab + high dose B-LAP (5 mg/kg/day); 5- Normal mice receiving 5 mg/kg/day B-LAP. Treatment doses of B-LAP were adopted according to a previously conducted similar study and the agent was administered via intra-gastric gavage (13). After 12 weeks of treatment, mice were housed in metabolic cages to collect 24-hour urine samples; and then, tissue and blood samplings were performed after appropriate anesthesia (50 mg/kg ketamine + 1 mg/kg midazolam).

***Measurement of systolic blood pressure***

The systolic blood pressure (SBP) readings were recorded a day before placing the mice in the metabolic cages using a non-invasive tail-cuff method (AD Instrument PowerLab Data Acquisition System, Australia). Animals were placed in a heated restrainer at 37 ± 1 °C for 10 min during measurements. For each mouse, 3 blood pressures were measured and their average was taken as the SBP.

***Biochemical and immunochemical assessments***

In order to calculate Ccr, first serum and urinary levels of creatinine were assayed via commercially available kits (Pars Azmoon, Tehran, Iran). Creatinine clearance (Ccr) was then calculated using the values for urinary creatinine, urine volume, serum creatinine, and body weight (14):

Ccr (mL/min/kg) = (urine creatinine (mg/dL) × urine volume (mL)) / (serum creatinine (mg/dL) × 1440 (min)) × 1000 / (body weight (g))

Renal malondialdehyde (MDA) and glutathione peroxidase (GPX) activities were measured spectrophotometrically using the commercially available kits (Biorex Fars, Shiraz, Iran). Obtained values were normalized by renal total protein levels measured by the Lowry method.

Tumor necrosis factor-alpha (TNF-α), nephrin, and neutrophil gelatinase-associated lipocalin (NGAL) levels were assayed by using enzyme-linked immunosorbent assay (ELISA) kits (EIAab, Wuhan, China).

***Histopathological examinations***

Renal tissue specimens were fixed in 10% neutral buffered formalin (NBF) and processed for paraffin sections of five-μm thickness. Sections were stained with hematoxylin and eosin (H&E) and were qualitatively examined under a light microscope (PW108, Proway, China).

For immunofluorescence analysis, five-µm kidney tissue sections were cut and incubated with antibodies raised against caspase-3 (Santa Cruz, sc-7272) after the antigens were retrieved enzymatically using 0.05% trypsin solution (Sigma) for 10 min. Subsequent to incubation with fluorescein isothiocyanate (FITC)-conjugated secondary antibody (Santa Cruz, sc-516140) solution, the slides were visualized using an immunofluorescence microscope (Olympus BX51, Tokyo, Japan). ImageJ software (version 1.41) was utilized to semiquantitatively analyze the data. For normalization, pixel intensities obtained from the treatment groups were divided by the corresponding pixel intensities recorded for the sham mice.

***Western blotting***

Sodium dodecyl sulfate-polyacrylamide gel electrophoresis was performed to electrophoretically separate the proteins. After the transfer of the separated proteins onto the PVDF membranes via electro-blotting, blocking was performed using a 5% skimmed milk solution for 60 min. Then, the membranes were incubated in caspase-3 (SantaCruz, sc-56053), phosphoinositide 3-kinase (PI3K) (SantaCruz, sc-1637), phosphorylated Akt (p-Akt) (SantaCruz, sc-136521), phosphorylated mammalian target of rapamycin (p-mTOR) (SantaCruz, sc-293133), phosphorylated cAMP response element-binding protein 1 (p-CREB-1) (SantaCruz, sc-81486), Sirtuin 1 (SIRT1) (SantaCruz, sc-74465), and β-actin (SantaCruz, sc-47778) primary antibodies at 4 °C overnight. Blots were then incubated by the horseradish peroxidase-labeled secondary antibody solution at room temperature for 45 min and were visualized by Western Blotting Luminol Reagent (Santa Cruz). ImageJ software (version 1.41) was implemented to measure the pixel intensities of the visualized bands from the X-ray films. The band density of each protein was first divided by the band density of its respective β-actin loading control and the obtained values for study groups were then normalized by the values for the sham group.

***Statistical analysis***

Data were descriptively expressed as mean ± SD. The variables were analyzed by one-way analysis of variance followed by Tukey’s test for *post hoc* comparisons using Statistical Package for the Social Sciences, (version 18.0, SPSS Inc., Chicago, USA). The statistical significance level was set at *P*<0.05.

## Results

General characteristics of the study groups have been presented in Table 1. Neither 2.5 mg/kg nor 5 mg/kg B-LAP had any significant effects on the body weights, blood glucose levels, serum lipid profile values, and SBP in the diabetic mice.

Renal TNF-α levels were significantly elevated in diabetic mice compared with the sham group [0.47 ± 0.052 pg/mg protein vs 0.18 ± 0.035 pg/mg protein; (*P*<0.01)]. TNF-α levels in the kidneys were declined by 23.4% after treatment with 5 mg/kg B-LAP (*P*<0.05) (Table 2).

Renal levels of MDA in diabetic mice were identified to be increased in diabetic mice (15.63 ± 1.22 nmol/mg protein) compared with sham mice (4.42 ± 0.92 nmol/mg protein) (*P*<0.01), and 5 mg/kg B-LAP significantly reduced renal MDA levels to 11.62 ± 1.19 nmol/mg protein (*P*<0.05) (Table 2).

Renal activities of the GPX enzyme in diabetic mice (13.71 ± 1.31 U/mg protein) were significantly higher than in sham mice (5.21 ± 1.06 U/mg protein) (*P*<0.01); and 5 mg/kg B-LAP significantly elevated its values to 15.62 ± 1.36 U/mg protein (*P*<0.05) in diabetic mice. It should be underlined that healthy mice receiving 5 mg/kg B-LAP had higher activities of GPX in their kidneys (7.46 ± 1.12 U/mg protein) compared with sham mice (5.21 ± 1.06 U/mg protein) (*P*<0.05) (Table 2).

Diabetic mice had higher levels of urinary nephrin in comparison with mice in the sham group [6.11 ± 0.95 µg/mg Cr vs 1.89 ± 0.35 µg/mg Cr; (*P*<0.01)]. Treatment with 5 mg/kg B-LAP reduced urinary levels of nephrin by 23.56% compared with diabetic mice (*P*<0.05). Urinary NGAL levels were significantly increased in diabetic mice (0.64 ± 0.073 ng/mg Cr) compared with the sham group (0.23 ± 0.042 ng/mg Cr) (*P*<0.01); and 5 mg/kg B-LAP reduced urinary NGAL levels to 0.53 ± 0.055 ng/mg Cr (*P*<0.05) (Table 2).

Creatinine clearance values were detected to be attenuated in diabetic mice (1.05 ± 0.12 mL/min/kg) in comparison with sham mice (2.21 ± 0.23 mL/min/kg) (*P*<0.01). Its values were increased to 1.44 ± 0.18 mL/min/kg after treatment with 5 mg/kg B-LAP (*P*<0.05) (Table 2).

**Figure 1:**
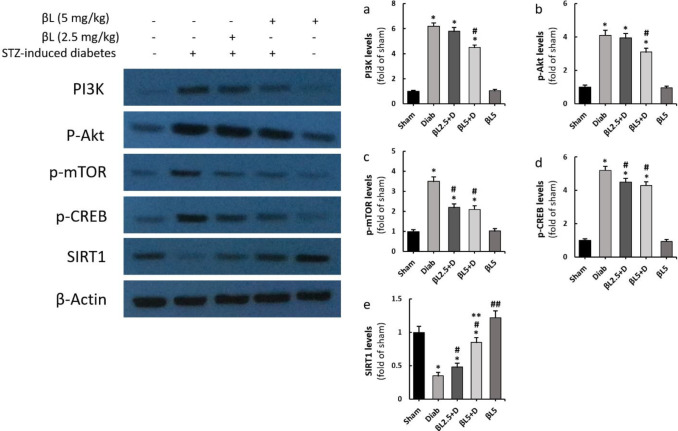
Effects of B-LAP on renal histopathology (400 ×); Micrographs of H&E stained sections of kidney; A, sham group (n = 7); B, control diabetic group (n = 7); C, diabetic mice treated with 2.5 mg/kg B-LAP (n = 7); D, diabetic mice treated with 5 mg/kg B-LAP (n = 7); E, normal healthy mice receiving 5 mg/kg B-LAP (n = 7). B-LAP, β-lapachone. (A) and (E) show that the renal histoarchitecture is normal. (B) and (C) demonstrate cell vacuolation (solid arrows), necrosis (arrow heads), cell detachment from underlying basement membrane (dotted arrows) pyknotic nuclei (chevrons), and glomerular retraction (stars). (D) shows that 5 mg/kg B-LAP alleviated renal histological derangements, but infrequent cell vacuolation (solid arrows) could still be observed

**Figure 2:**
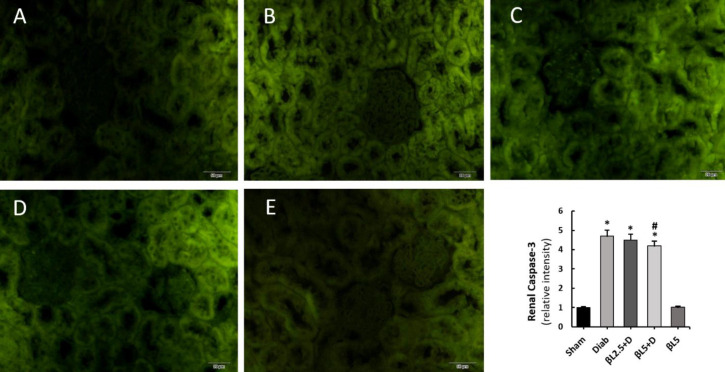
Effects of B-LAP on the renal expressions of cleaved caspase-3 as assessed by immunofluorescence microscopy. The relative fluorescence intensities of the diabetic mice were normalized by that of the normal control ones. Pixel intensities were calculated by using the ImageJ software (version 1.41) and the tests were done in triplicate. A, sham group (n = 6); B, control diabetic group (n = 6); C, diabetic mice treated with 2.5 mg/kg B-LAP (n = 6); D, diabetic mice treated with 5 mg/kg B-LAP (n = 6); E, normal healthy mice receiving 5 mg/kg B-LAP (n = 6). B-LAP, β-lapachone. ^*a^*P*<0.05 vs Sham: ^#^a*P*<0.05 vs Diab

Renal histopathological evaluations showed that 2.5 mg/kg B-LAP was almost ineffective in ameliorating kidney histoarchitecture and glomerular retraction as well as tubular cell detachment from underlying basement membrane could frequently be observed (Figure 1). By contrast, 5 mg/kg B-LAP successfully improved histopathological derangements in the kidneys of diabetic mice and only limited numbers of cell vacuolation could be identified (Figure 1). Additionally, renal levels of caspase-3 had significantly been elevated in the diabetic mice (4.7 fold of sham mice, *P*<0.01) and 5 mg/kg B-LAP slightly, albeit statistically significantly, attenuated its levels compared with control diabetic mice (*P*<0.05) (Figure 2).

**Figure 3:**
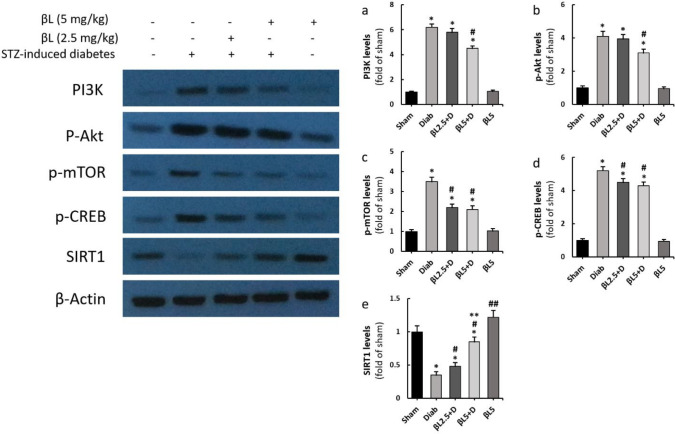
Effects of B-LAP on renal levels of PI3K (a), p-Akt (b), p-mTOR (c), p-CREB (d), and SIRT1 (e) as evaluated by western blotting. Sham, normal control group (n = 6); Diab, control diabetic group (n = 6); βL2.5+D, diabetic mice treated with 2.5 mg/kg B-LAP (n = 6); βL5+D, diabetic mice treated with 5 mg/kg B-LAP (n = 6); βL5, normal healthy mice receiving 5 mg/kg B-LAP (n = 6)

Renal levels of PI3K, p-Akt, p-mTOR, and p-CREB were all significantly elevated in diabetic mice in comparison with the sham group (*P*<0.01) (Figure 3). PI3K and p-Akt levels in the kidneys were only significantly decreased by 5 mg/kg B-LAP (Figures 3 a and b); on the other hand, both 2.5 mg/kg and 5 mg /kg doses of B-LAP were able to significantly mitigate renal levels of p-mTOR and p-CREB almost to the same extent (Figures 3 c and d). Conversely, renal levels of SIRT1 were recognized to significantly be reduced in diabetic mice and 2.5 mg/kg and 5 mg/kg B-LAP dose-dependently elevated its levels; furthermore, normal mice that received 5 mg/kg B-LAP had significantly higher levels of SIRT1 in their kidneys compared with sham mice (*P*<0.05) (Figure 3e).

## Discussion

The current study showed that B-LAP, a plant-derived naphthoquinone with potential anti-inflammatory activity, protected the kidneys against the deleterious effects of STZ-induced diabetes. In addition to improving renal function as demonstrated by elevated creatinine clearance values, B-LAP successfully declined renal and urinary levels of tubular injury markers, including nephrin and NGAL. Ameliorating renal histoarchitecture, B-LAP reduced renal levels of p-mTOR as well as p-CREB and at the same time increased SIRT1 levels in the kidneys of diabetic mice. 

B-LAP functions as the activator of NAD(P)H quinone dehydrogenase (NQO1), the enzyme that regenerates cellular NADH into NAD^+^; thereby, restored cellular NAD^+^ activates NAD^+^-dependent enzymes, including SIRT1 deacetylase. Deacetylated p65 subunit of NF-κB protein complex is prone to degradation by the ubiquitin-proteasome system (UPS); therefore, increased activity of SIRT1 suppresses inflammation by reducing the expression of cytokines that are downstream to NF-κB (15, 16). B-LAP not only dose-dependently up-regulated renal SIRT1 levels that were decreased due to DN but also raised SIRT1 levels in the kidneys of normal healthy mice. Similar findings have been obtained in cisplatin-induced nephrotoxic mice treated with B-LAP (17). In line with these findings, B-LAP up-regulates the cardiac concentrations of SIRT1 and Nrf2 in doxorubicin-induced cardiotoxic mice that apart from alleviating inflammatory cytokines, reduce cardiac distress markers and improve heart function (18).

Our findings showed that B-LAP especially at 5 mg/kg attenuated elevated levels of PI3K, p-Akt, p-mTOR, and p-CREB in the kidneys of diabetic mice. The induction of the PI3K/Akt/mTOR signaling pathway contributes significantly to the pathogenesis of DN as it initiates the pathways involved in kidney fibrosis and inflammation (19). In agreement with the previous findings, we noticed reductions in the renal levels of TNF-α, the salient pro-inflammatory cytokine (20), in 5 mg/kg B-LAP receiving diabetic mice. The reductions in TNF-α levels were accompanied by significant amelioration in kidney histoarchitecture in the mice that received 5 mg/kg B-LAP. As reported by Wu *et al*., Huang Kui capsule (HKC) alleviates glomerular and tubular pathological changes associated with DN and simultaneously represses renal levels of p-Akt and p-mTOR in mice (21). Likewise, Huang *et al*., stated that qufengtongluo (QFTL), in addition to repressing renal levels of PI3K and p-AKT, down-regulates transforming growth factor-beta (TGF-β) expressions in the kidneys and ameliorates albuminuria in type 2 diabetic rats (22).

CREB is a transcription factor that after phosphorylation is translocated into the nuclei of the renal cells and functions as the regulator of the expression of genes encoding several pro-fibrotic (23) and antioxidant proteins (24). We found elevated levels of p-CREB in the renal tissues of diabetic mice that were attenuated by both 2.5 mg/kg and 5 mg/kg of B-LAP. All tested variables in the present investigation were only effectively alleviated by 5 mg/kg B-LAP, except for p-CREB and p-mTOR, levels of which both low and high doses of the agent attenuated almost equally. This observation could be explained by the fact that the activation/inhibition of these two downstream proteins is regulated by several disparate factors, including AMPK (23), ERK (25), glucose-stimulated USF2 up-regulation (26), and angiotensin II receptor-induced PKA activation (27).

Demonstration of reduced MDA levels and elevated GPX activities in the renal tissues by B-LAP treatment is an indication of ameliorations in oxidative stress, the indispensable feature of DN both in mice and in humans (28). Likewise, dunnione, a B-LAP analogue, is able to up-regulate the activity of antioxidant enzymes and attenuate MDA levels in the kidneys of cisplatin-induced nephrotoxic rats via promoting the nuclear presence of Nrf2, the master regulator of antioxidant response (29). It is well known that SIRT1 directly activates Nrf2 (30), and the observed enhancement in GPX activities with simultaneous reductions in MDA levels in the kidneys could have possibly been mediated via this mechanism.

Glomerular basement membrane disintegration, as well as podocyte detachment, are two principal pathogenic features of DN (31). 5 mg/kg B-LAP mitigated increased urinary levels of nephrin in the diabetic mice. Nephrin is a fibrillar protein, specifically expressed by the glomerular podocytes, that spans the foot process and participates in the formation of the glomerular filtration barrier (32). Moreover, elevated urinary excretions of NGAL were ameliorated by 5 mg/kg B-LAP treatment. NGAL is an intracellular protein, specifically expressed in the renal tubular epithelial cells (33). Therefore, it could be inferred that B-LAP renders protection against both glomerular podocytes and tubular epithelial cells in the kidneys. In line with these findings, kidney histoarchitecture and caspase-3 relative fluorescent intensity were significantly ameliorated by 5 mg/kg B-LAP.

**Table 1:** General characteristics of mice in the study groups

	Sham (n = 6)	Diab (n = 6)	βL2.5+D (n = 6)	βL5+D (n = 6)	βL5 (n = 6)
**Body weight (g)**	28.5 ± 1.5	23.6 ± 1.7^a^	23.1 ± 1.9^a^	23.8 ± 1.2^a^	23 ± 1.5^a^
**Blood glucose (mg/dL)**	85 ± 7	382 ± 38^a^	396 ± 45^a^	390 ± 36^a^	379 ± 48^a^
**Serum TG (mg/dL)**	131 ± 9	224 ± 13^a^	219 ± 11^a^	218 ± 15^a^	226 ± 10^a^
**Serum cholesterol (mg/dL)**	76 ± 7	111 ± 9^a^	115 ± 8^a^	116 ± 11^a^	118 ± 9^a^
**Serum HDL-C (mg/dL)**	55 ± 3	32 ± 4^a^	30 ± 5^a^	32 ± 5^a^	33 ± 3^a^
**Serum LDL-C (mg/dL)**	149 ± 15^a^	248 ± 13^a^	257 ± 10^a^	251 ± 12^a^	245 ± 11^a^
**SBP (mmHg)**	125 + 8	127 ± 5	122 ± 7	125 ± 10	123 ± 8

Sham, normal control group. Diab, control diabetic group. βL2.5+D, diabetic mice treated with 2.5 mg/kg β-Lapachone. βL5+D, diabetic mice treated with 5 mg/kg β-Lapachone. βL5, normal healthy mice receiving 5 mg/kg β-Lapachone.

TG: triglyceride; HDL-C: high-density lipoprotein cholesterol; LDL-C: low-density lipoprotein cholesterol; SBP: systolic blood pressure

^a^ *P*<0.01 vs Sham

**Table 2:** Markers of renal inflammation, oxidative stress, kidney damage, and renal function in mice in the study groups

	Sham (n = 6)	Diab (n = 6)	βL2.5+D (n = 6)	βL5+D (n = 6)	βL5 (n = 6)
**TNF-α** **(pg/mg protein)**	0.18 ± 0.035	0.47 ± 0.052^a^	0.44 ± 0.041^a^	0.36 ± 0.046^a,b^	0.17 ± 0.032
**MDA** **(nmol/mg protein)**	4.42 ± 0.92	15.63 ± 1.22^a^	14.75 ± 1.14^a^	11.62 ± 1.19^a,b^	4.63 ± 0.95
**GPX** **(U/mg protein)**	5.21 ± 1.06	13.71 ± 1.31^a^	14.14 ± 1.33^a^	15.62 ± 1.36^a,b^	7.46 ± 1.12^a^
**Urine nephrin** **(µg/mg Cr)**	1.89 ± 0.35	6.11 ± 0.95^a^	5.88 ± 0.79^a^	4.67 ± 0.66^a,b^	1.98 ± 0.41
**Urine NGAL** **(ng/mg Cr)**	0.23 ± 0.042	0.64 ± 0.073^a^	0.61 ± 0.068^a^	0.53 ± 0.055^a,b^	0.22 ± 0.038
**Creatinine clearance** **(mL/min/kg)**	2.21 ± 0.23	1.05 ± 0.12^a^	1.12 ± 0.14^a^	1.44 ± 0.18^a,b^	2.18 ± 0.24

Sham, normal control group. Diab, control diabetic group. βL2.5+D, diabetic mice treated with 2.5 mg/kg β-Lapachone. βL5+D, diabetic mice treated with 5 mg/kg β-Lapachone. βL5, normal healthy mice receiving 5 mg/kg β-Lapachone.

C:, creatinine; GPX: glutathione peroxidase; MDA: malondialdehyde; NGAL: neutrophil gelatinase-associated lipocalin; TNF-α: tumor necrosis factor-alpha

^a^ *P*<0.01 vs Sham; ^b^*P*<0.05 vs Diab

B-LAP: beta-lapachone

^*a^*P*<0.01 vs Sham; ^#a^*P*<0.05 vs Diab; ^##a^*P*<0.05 vs Sham; ^**a^*P*<0.05 vs βL2.5+D

## Conclusion

Our findings underline the protective actions of B-LAP on the renal complications of STZ-induced diabetic mice. Apart from reductions in the urinary indices of glomerular and tubular injury, creatinine clearance values, the indicators of renal function, were significantly improved in diabetic mice by one-month B-LAP treatment. Moreover, 5 mg/kg B-LAP confers protection against renal inflammation and oxidative stress in STZ-induced diabetic mice probably via modulating the activities of PI3K/Akt/mTOR and SIRT1 levels in the kidneys.
